# Biomimetic Studies on the Antimicrobial Activity of Some Biocides Based on Garlic and Lavender in Surface Waters

**DOI:** 10.3390/biomimetics9100591

**Published:** 2024-09-29

**Authors:** Mădălina Grinzeanu, Oanamari Daniela Orbuleț, Annette Madelene Dăncilă, Constantin Bobirică, Cristina Modrogan, Liliana Bobirică, Mădălina Andreea Pandele

**Affiliations:** Faculty of Chemical Engineering and Biotechnology, National University of Science and Technology Politehnica of Bucharest, 1-7 Gheorghe Polizu Street, 011061 Bucharest, Romania; madelene.dancila@upb.ro (A.M.D.); constantin.bobirica@upb.ro (C.B.); cristina.modrogan@upb.ro (C.M.); liliana.bobirica@upb.ro (L.B.); madalina.pandele@upb.ro (M.A.P.)

**Keywords:** biomimetics, pathogen agents, aquatic ecosystems, antimicrobial activity, biocides, garlic, lavender

## Abstract

For a given aquatic ecosystem that will be used as a water source, it is necessary to establish the quality of the water from a microbiological point of view by identifying the pathogens present in the water. The aim of this study was to determine and analyze the antimicrobial activity of some biocides derived from garlic (garlic–methanol extract) and lavender (lavender–water extract). Their efficiency was evaluated at different concentrations and contact times. Initially, through specific laboratory analyses, the microbiological characteristics of the river were determined. Biomimetic studies on the antimicrobial activity of biocides based on garlic and lavender in surface waters involved detailed exploration of how the natural antimicrobial properties of these plants can be effectively utilized to treat water contaminated with harmful microorganisms. Both the contact time and the amount of biocide used have a significant effect on the microorganisms of interest. Thus, to describe the degradation rate of coliform bacteria, a pseudo-first-order and zero-order kinetic model was used, $r=-(dN/dt)=k_{obs}\cdot t$ şi $r_{0}=k_{obs}\cdot N_{0}=k_{0}$, where r is the rate of degradation of microorganisms (CFU/min), N_0_ is the initial number of microorganisms in the aqueous solution (colony-forming unit, CFU), N is the final number of microorganisms after a contact time t (CFU), k_obs_ is the pseudo-first-order rate constant (min^−1^), t is the contact time (min), r_0_ is the initial rate of degradation of microorganisms (CFU/min), and k_0_ is the pseudo-rate constant zero order (min^−1^). Following 60 min of treatment with 1 mL of lavender-water biocide, the inhibition rate of pathogenic microorganisms in the water reached 59.09%, whereas, under the same conditions, the garlic–methanol biocide achieved an inhibition rate of 40.86%. This study confirms the antimicrobial activity of both lavender and garlic biocides, highlighting their potential in mitigating water pollution caused by pathogens.

## 1. Introduction

Being used for various purposes, water is essential for life. Many health problems are caused by the unavailability or low quality of drinking water. Drinking water sources typically come from groundwater or surface water, with less frequent use of alternative sources, such as seawater. Coliform bacteria are commonly found in aquatic environments and soil, because they are universally present, in large quantities, in human waste and in the feces of warm-blooded animals. Surface waters often contain pathogenic microorganisms due to specific sources of pollution (livestock farms, urban sewage treatment plants) and diffuse sources (agricultural soils, rainwater, etc.) [1].

Incorporating garlic- and lavender-based biocides into water treatment processes can help reduce microbial contamination in surface waters, improving water quality and safety. Additionally, these biocides can be applied as coatings to surfaces that come into contact with water, such as pipelines and storage tanks, to prevent biofilm formation and microbial growth [2].

The rivers flowing through intensely populated areas are reported to be contaminated with fecal coliform (e.g., *Escherichia coli*, *enterococci bacteria*) [3,4], enteric viruses [4,5,6,7], and protozoa (e.g., *Cryptosporidium* spp. and *Giardia duodenalis*) [8]. Atoyan et al. (2011) evidenced the presence of *Bifidobacterium adolescentis* DNA, which is an indicator of river contamination by human fecal coliforms [3]. Regardless of its origin (animal or human), fecal pollution of water rivers can be a source of pathogenic bacteria, viruses, protozoa, and helminths. It has been shown that the surface water quality depends on several factors that are interconnected: degree of urbanization of the region, existence of farming activities, anthropogenic utilization of the river (e.g., recreation, transportation that can be sources of contamination by fecal pathogens), and seasonal changes [6,9,10,11]. All these factors combine and influence the transport of pathogens in the freshwaters. Contamination by *Escherichia coli* was found to be elevated on Manso River (Brazil) and it was attributed to the discharge of insufficiently treated domestic sewage [12]. The significant pollution by fecal pathogens in the Danube River as it passes through several European countries has been linked to the existence of large urban municipalities [11]. Wastewater treatment plants were found to reduce the number of pathogens in the main river, while tributary rivers were identified as contributing to increased microbial pollution [8]. Untreated wastewater discharges are associated with the persistent fecal contamination of rivers [13].

As indicators of fecal contamination of a water body, picobirnavirus (PBV), human adenovirus (HAdV), and infective enterovirus (iEV) have been proposed [4,14]. It has been reported that only looking at the classical indicators of *Escherichia coli* or enterococci will not offer any insight into viruses’ presence in the water body [5,8].

Due to the alarming increase in antimicrobial resistance when using the available synthetic drugs, attention has shifted towards plant extracts known to have antibiotic properties. Other plants or spices that have been reported to have enhanced antimicrobial properties are cinnamon, garlic, onion, oregano, ginger, coriander, and rosemary [15,16,17]. More recent studies have also revealed the antimicrobial properties of lavender, particularly in cosmetic applications. Among the plants with notable antimicrobial properties, garlic and lavender are of particular interest [18,19].

The use of biopesticides as an alternative to synthetic pesticides has gained popularity due to the growing awareness of the harmful effects of chemicals on the environment and human health. Biopesticides provide an environmentally friendly solution by using plant extracts to manage pest populations. Synthetic products have been widely used for agricultural pest control, but their extensive use has led to serious environmental and public health problems, including soil and water pollution, toxicity to non-target organisms, and the development of resistance. These concerns have driven interest in more sustainable alternatives like biopesticides. Allicin (diallyl thiosulfinate), a sulfur-containing compound, is the most abundant component of garlic and has been extensively studied for its antimicrobial and antioxidant properties [20]. Allicin is produced by garlic tissues as a defense against pathogens and pests, functioning as a reactive sulfur species that oxidizes accessible cysteines from glutathione and proteins [21]. Allicin acts as a biocide with a broad spectrum of activity against a wide range of microorganisms [21,22].

Biomimetic studies aim to replicate natural processes and systems to develop innovative solutions. In the context of antimicrobial activity, such research often draws inspiration from natural substances with known antimicrobial effects, like garlic and lavender, applying them to areas such as water treatment. The primary antimicrobial agent in garlic is allicin, which is released when garlic is crushed or chopped and extracted with solvents. Allicin disrupts microbial cell membranes and inhibits essential enzyme functions. Similarly, essential oils from lavender, especially compounds like linalool and linalyl acetate, have demonstrated antimicrobial properties by penetrating microbial cell membranes and interfering with cellular processes [2].

Allicin, a key active compound in freshly crushed garlic, exhibits broad antimicrobial activity. It has been shown to be effective against a variety of Gram-negative and Gram-positive bacteria, including multidrug-resistant strains of *Escherichia coli*. Additionally, allicin has antifungal properties, particularly against *Candida albicans*, as well as antiparasitic effects on intestinal parasites like *Entamoeba histolytica* and *Giardia lamblia*. It also displays antiviral activity against pathogens such as *E. histolytica* [22].

Lavender has also been reported to have antimicrobial effects, in addition to its antioxidant and anti-inflammatory properties [23]. The phenolic acids, flavonoids, and terpenoids in lavender contribute to its extensive use in traditional remedies [24]. It has been demonstrated that lavender essential oil is effective in reducing *Staphylococcus species* in a hospital environment [25]. *Salmonella*, *Enterobacter*, *Klebsiella*, *E. coli*, *Listeria monocytogenes*, *P. aeruginosa*, *S. aureus*, *B. subtilis A. niger*, *P. notatum* and *C. albicans*, *Bacillus subtilis*, *S. aureus,* and Gram-negative bacteria and *Listeria monocytogenes* are all inhibited by lavender extract [23].

Like garlic, the composition of the compounds of interest varies among the lavender species and depends on the harvesting location [26].

This study aims to investigate the removal efficiency of fecal coliform from surface waters with the help of garlic and lavender extracts. The broad spectrum of antimicrobial activity, along with the low toxicity of garlic and lavender, are properties that make these plants suitable for developing derived products to treat water against certain pathogens that threaten human health.

## 2. Materials and Methods

Through specific laboratory analyses, the microbiological characteristics of coliform bacteria in the river were determined, and the influence of certain biocides on the coliform bacteria present in the surface water was evaluated. The samples were collected in sterile vials that were appropriately labeled (including date of sampling, type of water, name of the samples).

Before beginning the experimental testing of the biocides, an analysis of potential influencing factors was conducted. It was found that the pH of the water source does not vary significantly, while the temperature does not affect the efficiency of the biocides but influences the number of developing microorganisms.

### 2.1. Procedure for the Testing of Microorganisms’ Removal from Surface Waters

The city of Bucharest, the capital of Romania, uses water from the Arges river for its drinking water supply. Since coliform bacteria pose a significant health risk to the population, this study aimed to remove them using plant extracts. Chlorine gas, typically used in treatment plants, is toxic to the human body; therefore, we sought to replace it with these plant extracts. Water samples were taken at a depth of 30 cm below the water surface.

The characterization of the initial water used for disinfection with biocides is presented in Table 1. The water used for disinfection was natural water from the Arges River, Romania.

Counting analyses of coliform bacteria were conducted in the laboratory using the membrane filtration method (according to SR EN ISO 9308-1: 02.2025). A specific volume of surface water (e.g., 1000 mL) and the desired amount of biocide (1–10 mL) were added to a sterile test tube and mixed for 15, 30, or 60 min. The sample was homogenized by mechanical stirring at 350 rpm/min using a 6-position magnetic stirrer, MULTI STIRRER Digital VELP (VELP Scientifica, Usmate Velate, Italy) for the designated contact time, then filtered through a membrane with a nominal pore size of 0.45 µm using a filtration setup. Then, it was placed in a 55 mm diameter Petri dish containing CCA medium (Chromogenic Coliforms Agar—Biolife Italiana, Milan, Italy) and incubated at 36 ± 2 °C, for 21–24 h with an incubator Steinberg SBS-LI-18. After the incubation period, the samples were examined using an A SKU 0104 microscope with magnification between 40× and 1600×.

A control sample was included for every set of experiments testing the antimicrobial activity of the biocides; for the control sample, the procedure was identical to the actual samples, but no disinfectant was added.

### 2.2. Garlic- and Lavender-Based Biocide Synthesis

#### 2.2.1. Garlic-Based Biocide Synthesis

A fresh plant–methanol extract was prepared in a mass ratio of 1:5. Fresh natural products, purchased from the agricultural food markets, were used for the preparation of the biocide. The cleaned garlic bulbs were weighed (100 g) and finely chopped to obtain a homogeneous consistency. The mechanically crushed samples were mixed continuously with the appropriate solvent (methanol) for 24 h. After 24 h of mixing, the samples were filtered and preserved for characterization and preliminary testing of the antimicrobial activity.

#### 2.2.2. Lavander-Based Biocide Synthesis

A fresh plant–water extract was prepared in a mass ratio of 1:5. Fresh natural products, purchased from the agricultural food markets, were used for the preparation of the biocide. The cleaned garlic bulbs (100 g) were finely chopped to achieve a uniform consistency. The crushed samples were then subjected to continuous mechanical mixing for 24 h in water as the solvent. After 24 h of mixing, the samples were filtered and stored for subsequent characterization and preliminary antimicrobial activity testing.

The solvents used for the preparation of the extracts were distilled water (prepared in our own laboratories) and methanol. The high-purity methanol used for the biocide synthesis was provided by REDOX Research and Analytic.

GC-MS analysis was performed using an Agilent 6890 gas chromatograph coupled to an Agilent 5973 N mass spectrometer (Agilent Technologies, Santa Clara, CA, USA). For the component separation, an HP − 5MS GC column with the following characteristics was used: 30 m × 0.32 mm (internal diameter), substrate with a thickness of 25 μm. The carrier gas was helium, with a flow rate set to 1.5 mL/min, and the injector temperature was maintained at 260 °C. The heating temperature was set in the range of 60–260 °C, with a heating rate of 5 °C/min. The electron impact ion source was kept at 200 °C, and mass spectra were recorded at 70 keV. The identification of the components was conducted in scanning mode using the NIST11 and Wiley8 library.

FTIR spectra were recorded on a Bruker VERTEX 70 spectrometer (Bruker Corporation, Ettlingen, Germany) using 32 scans with a resolution of 4 cm^−1^ in the wavenumber range of 4000–600 cm^−1^.

NMR spectra (^1^H NMR and ^13^C NMR) were recorded on a Bruker Avance III 400 instrument ((Bruker Corporation, Ettlingen, Germany), operating at 400.13 MHz for ^1^H and 100.63 MHz for ^13^C. The samples were analyzed using either a 5 mm direct-sensing four-nuclei z-gradient probe or a 5 mm multinuclear reverse-sensing z-gradient probe. The spectra of the extracts were recorded with the NOESY preset pulse sequence. Analytical signals are reported as δ values (ppm) relative to TSP (sodium trimethylsilyl propionate), which is used as an internal standard (0.00 ppm).

## 3. Results

The antimicrobial activity was evaluated by exposing the water sample to various volumes of biocide, ranging from 1 to 10 mL, with different contact times of 1 to 60 min. The experiments were conducted in triplicate, and the results showed standard deviations of less than 10% of the average measurements.

Based on the laboratory analyses and the results obtained, graphs and tables were created to illustrate the effectiveness of the biocides in eliminating the targeted microorganisms, specifically coliform bacteria.

**Figure 1:**
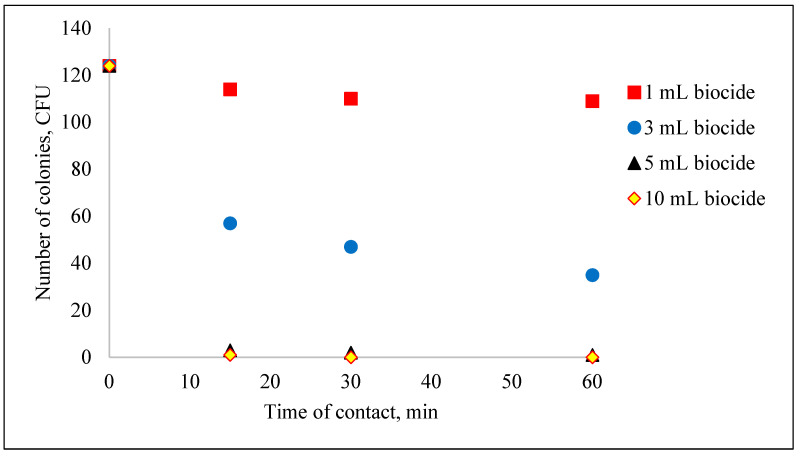
The decrease in the concentration of microorganisms after the addition of the garlic–methanol biocide.

**Figure 2:**
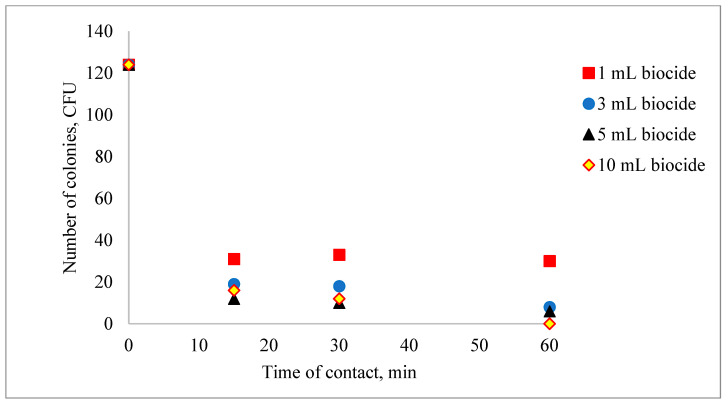
The decrease in the concentration of microorganisms after the addition of the lavender–water biocide.

The influence of the biocide concentration on the destruction of coliform bacteria is shown in Figure 1 and Figure 2. It can be observed that the efficiency of removing fecal coliforms increases with the amount of biocide used. However, to ensure economic feasibility, it is recommended to use a volume of 3 mL of biocide per 1000 mL of water.

In Figure 1, as the volume of biocide and the contact time increase, the concentration of the microorganisms of interest decreases significantly. A total inhibition of pathogenic microorganisms in the water occurred after adding a volume of 5 mL for 60 min, achieving a yield of 100%.

In Figure 2, as the volume of biocide and the contact time increase, the concentration of coliform bacteria in the water decreases. A total inhibition of coliform bacteria is observed after adding a volume of 10 mL for 60 min.

The biocides were analyzed to identify chemical compounds with antimicrobial activity. Therefore, they underwent GS-MS (gas chromatography coupled to mass spectrophotometry), FTIR (Fourier-transform infrared spectrophotometry), and NMR (nuclear magnetic resonance) analysis.

### 3.1. GC-MS Analysis of the Extract

**Figure 3:**
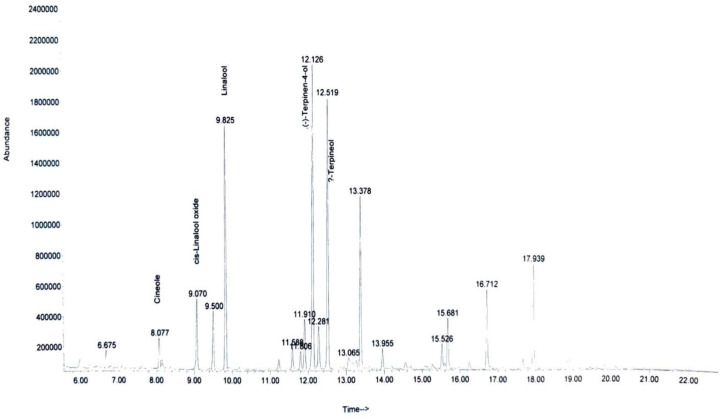
GC-MS spectrum of lavender–water extract.

The GC-MS analysis of the green biocides subjected to characterization indicated the presence of compounds recognized for their antimicrobial properties. Specifically, organic sulfur compounds such as 2-vinyl-4H-1,3-dithiine,3-vinyl-1,2-dithiacyclohex-4(5)-ene and di-2-propenyl trisulfide were identified in the garlic extracts, all of which possess antimicrobial activity. Two major chemical compounds identified in the lavender extracts were linalool and cineole, both recognized for their antimicrobial properties (Figure 3).

**Figure 4:**
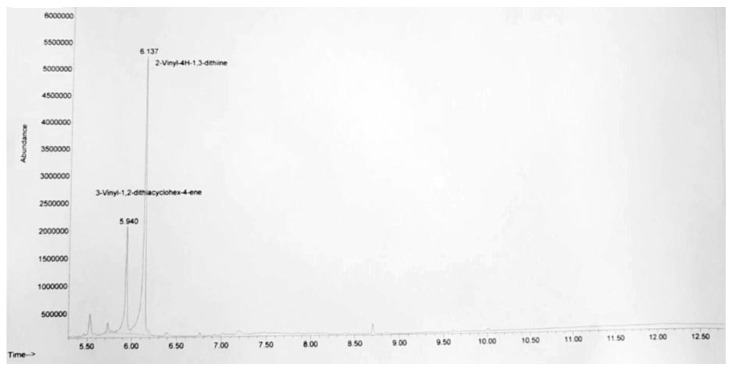
GC-MS spectrum of garlic–methanol extract.

The GC-MS analysis of the garlic–methanol mixture allowed for the identification of compounds known to have antimicrobial activity (Figure 4).

The compounds identified by GC-MS analysis were confirmed by FTIR and NMR analysis. Although some extracts revealed the presence of other organic compounds from the natural products used in the extraction, the dominant analytical signals identified by GC-MS indicate that the primary components are the green biocides that were prepared.

### 3.2. FTIR Analysis of the Extract

**Figure 5:**
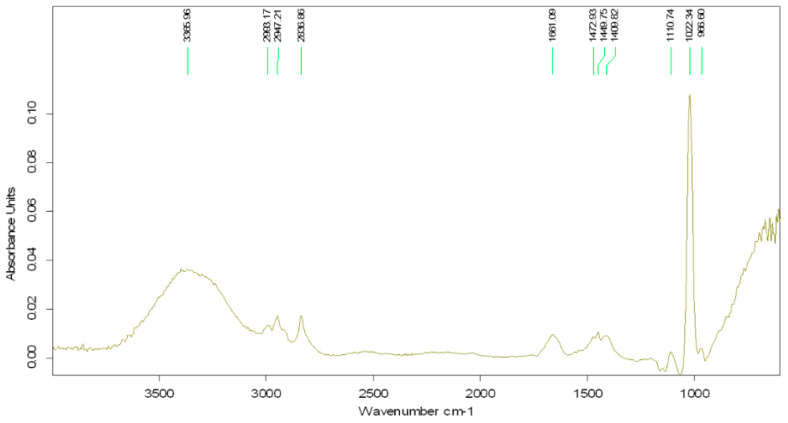
FTIR spectrum of garlic–methanol extract.

The FTIR spectrum of the garlic–methanol extract (mass ratio 1:5) shows a clear signal at the wavelength of 1022.34 cm^−1^ and a weaker signal at 1110.74 cm^−1^, both attributed to the extraction solvent, methanol (Figure 5). The broad peak at 3365.96 cm^−1^ can be attributed to the stretching vibration of the O-H bond in hydroxyl groups in aliphatic compounds. The peak at 2947.21 cm^−1^ is assigned to C-H groups in aliphatic compounds. The peak at 1661.09 cm^−1^ corresponds to the C=C double bond in the allyl group, while the peak at 2836.86 cm^−1^ is associated with the CH_2_ group in aliphatic compounds.

**Figure 6:**
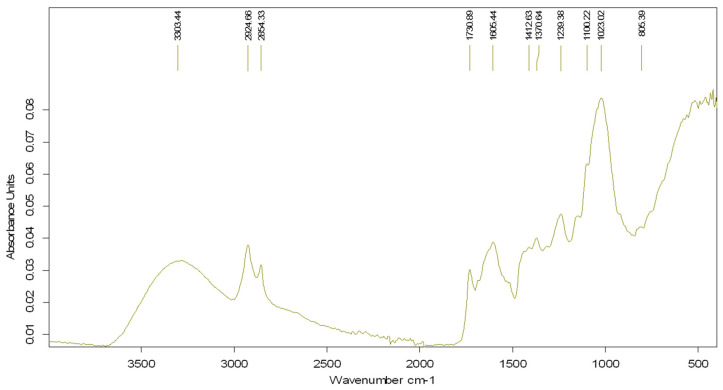
FTIR spectrum of lavender–water extract.

Figure 6 shows the FTIR spectrum of the lavender–water extract. The peak at the wavelength of 1023.02 cm^−1^ is attributed to the solvent, methanol. The peak at 1239.38 cm^−1^ may be assigned to C–O stretching vibrations while the peak at 1370.89 cm^−1^ could be attributed to the C–H bending vibration [27]. The peak at the wavelength of 1412.63 cm^−1^ can be attributed to the CH deformation vibration of methyl and methylene groups in lavender plants [28]. This peak is characteristic to linalool and linalyl acetate, which are the main constituents of lavender plants [29,30].

### 3.3. NMR Analysis of the Extract

**Figure 7:**
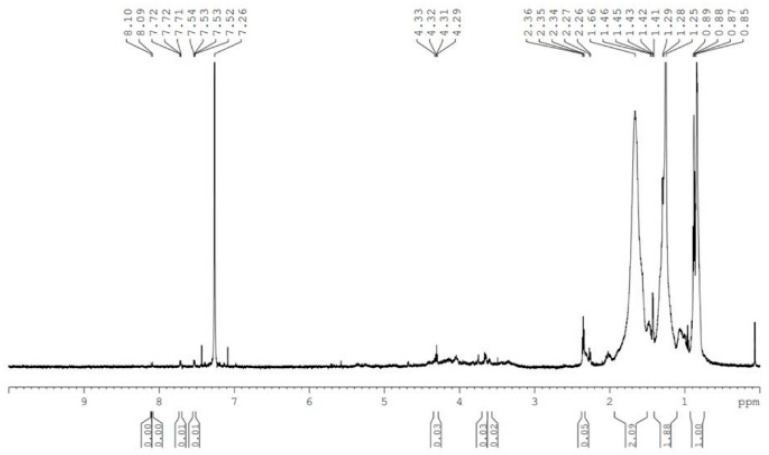
^1^H NMR spectrum of garlic–methanol extract.

In the ^1^H NMR spectrum of the garlic–methanol extract, the group of signals recorded in the range 4.33–4.29 ppm indicates the presence of organic compounds containing sulfur (Figure 7).

Allicin has the formula C_6_H_10_OS_2_. This was highlighted with the use of NMR.

The ^13^C NMR spectra of both the garlic–water and garlic–methanol extracts are inconclusive.

### 3.4. Degradation Kinetics

To describe the rate of degradation of coliform bacteria, a pseudo-first-order kinetic model was employed by graphically representing ln(N_0_/N) as a function of the contact time of the lavender–water or garlic–methanol biocides. This empirical model has been previously used to fit the experimental data on microorganism degradation [31,32,33]. The pseudo-first-order kinetic model and its linearized form are given by Equations (1) and (2):

(1) $$r=-\frac{dN}{dt}=k_{obs}\cdot t$$

(2) $$ln\left(\frac{N_{0}}{N}\right)=k_{obs}\cdot t$$

where r is the rate of degradation of microorganisms (CFU/min), N_0_ is the initial number of microorganisms in the aqueous solution (colony-forming unit, CFU), N is the final number of microorganisms after a contact time t (CFU), k_obs_ is the pseudo-first-order rate constant (min^−1^), and t is the contact time (min). The k_obs_ constant is determined from the slope of the linearized model (Figure 5).

After 60 min (in the case of the lavender–water biocide) and after 5 min (for garlic–methanol biocide) of contacting the sample, a decrease in the content of microorganisms of 96% and 99.99%, respectively, is achieved. It was assumed that the slope interval corresponding to a lower degradation rate describes the degradation of pathogenic microorganisms. Conversely, if this does not occur, it is assumed that the degradation follows pseudo-zero-order kinetics. The initial rate of degradation of coliform bacteria should be approximately the same for any initial concentration of them. To test this hypothesis, the initial rate of the degradation process is calculated assuming pseudo-zero-order kinetics.

(3) $$r_{0}=k_{obs}\cdot N_{0}=k_{0}$$

where r_0_ is the initial rate of degradation of microorganisms (CFU/min), k_obs_ is the observed pseudo-first-order rate constant (determined from the pseudo-first-order kinetic model), (min^−1^) and k_0_ is the pseudo-rate constant zero order (min^−1^). The kinetic parameters for the degradation of pathogenic microorganisms using the two types of biocides are presented in Table 2 and Table 3.

From the data presented in Table 2 and Table 3, it can be concluded that the computed data generally fit well with the experimental data (with R^2^ values higher than 0.94), except for the experiments conducted with the smallest volume of biocide (1 mL for lavender–water and 5 mL for garlic–methanol biocide).

**Figure 8:**
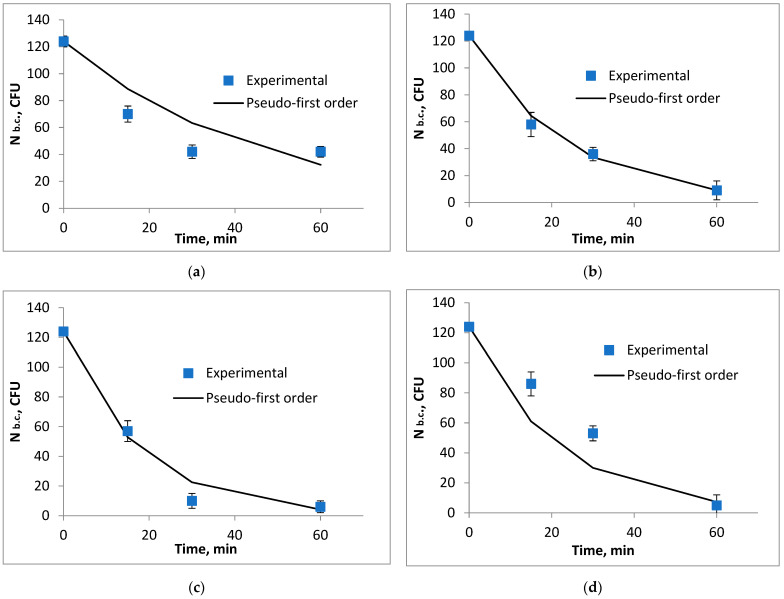
Variation in the number of coliform bacteria depending on the contact time with the lavender–water biocide, (**a**) V = 1 mL, (**b**) V = 3 mL, (**c**) V = 5 mL, (**d**) V = 10 mL.

**Figure 9:**
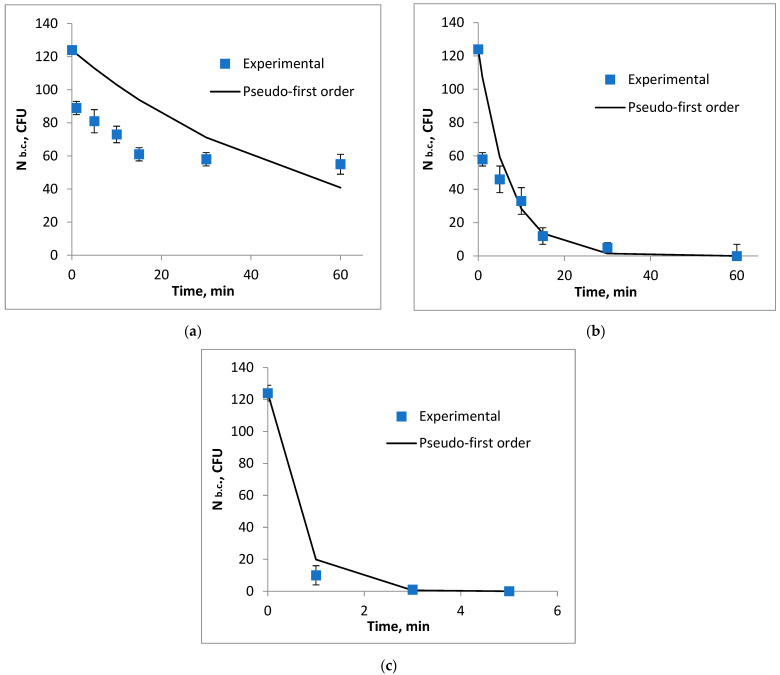
Variation in the number of fecal coliforms depending on the contact time with the garlic–methanol biocide, (**a**) V = 1 mL, (**b**) V = 5 mL, (**c**) V = 10 mL.

**Figure 10:**
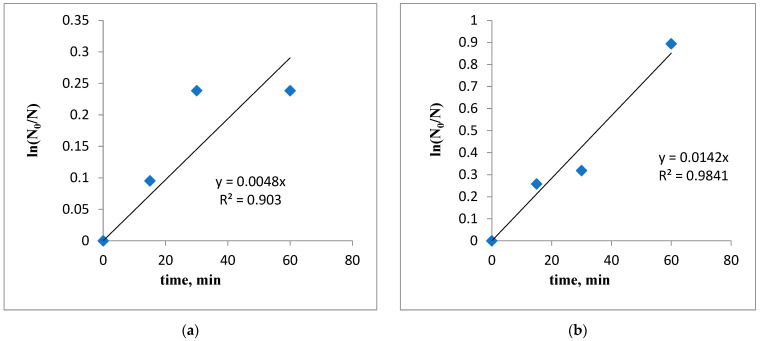

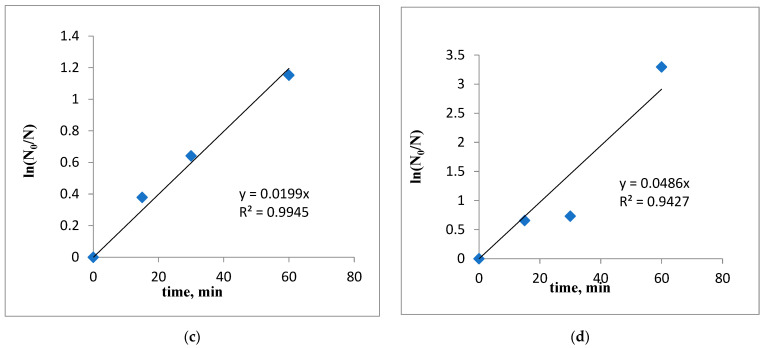
Pseudo-first-order kinetic model for the degradation of coliform bacteria in contact with lavender–water biocide, (**a**) V = 1 mL, (**b**) V = 3 mL, (**c**) V = 5 mL, (**d**) V = 10 mL.

The initial rate of degradation of coliform bacteria, r_0_, decreases as the volume of the biocidal agent decreases (Figure 8, Figure 9 and Figure 10).

The data were statistically analyzed using one-way analysis of variance (one-way ANOVA) at a significance level of α = 0.05 by using OriginPro 8 software (Table 4, Table 5, Table 6 and Table 7).

The ANOVA test results for lavender–water and garlic–methanol biocides at different volumes (1 mL and 10 mL) indicate significant differences between the groups in most cases. The high F values and extremely low *p* values (*p* < 0.05) suggest that the biocides had distinct effects on coliform bacteria, depending on the volume and type of biocide used. These results demonstrate that both the type of biocide and the volume used have a significant impact on the effectiveness in eliminating coliform bacteria.

As shown, the pseudo-first-order rate constant increases from k_obs_ = 0.0048 min^−1^ to k_obs_ = 0.0486 min^−1^, and the initial rate of degradation of microorganisms, r_0_, also rises with the increase in the volume of lavender–water biocide (Figure 10).

**Figure 11:**
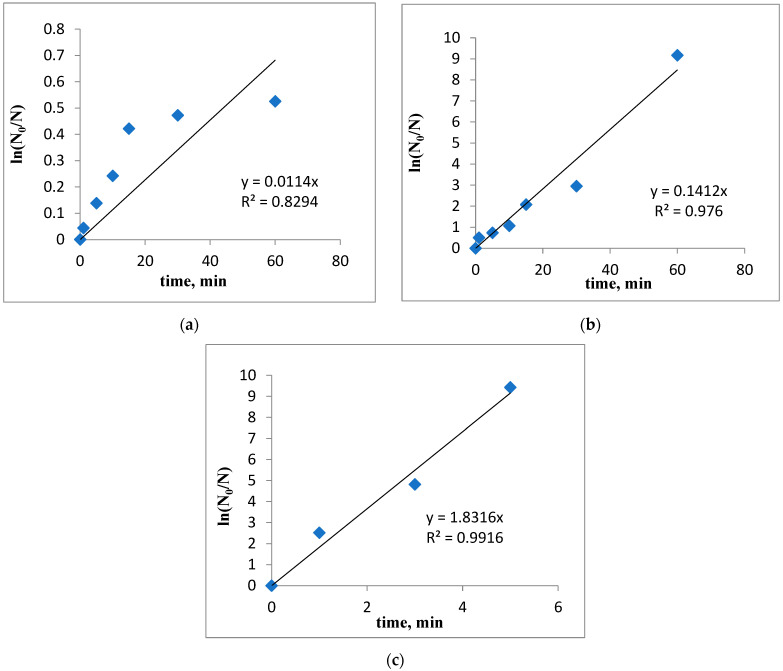
Pseudo-first-order kinetic model for the degradation of fecal coliforms in contact with garlic–methanol biocide, (**a**) V = 1mL, (**b**) V = 5 mL, (**c**) V = 10 mL.

For the garlic–methanol biocide, the pseudo-first-order rate constant increases from k_obs_ = 0.0114 min^−1^ to k_obs_ = 1.8316 min^−1^. Accordingly, the initial rate of degradation of microorganisms, r_0_, also increases with the volume of biocide, ranging from 1mL to 10 mL (Figure 11).

The degree of degradation for pathogenic microorganisms in the presence of the lavender–water biocide increases with the contact time, exceeding 96% after 60 min (Table 8). A reduction by half in the number of coliform bacteria occurs after approximately 30 min with biocide volumes of 3 mL or 5 mL, and after about 60 min with a biocide volume of less than 1 mL. The increase in the biocide concentration is a favorable aspect in the destruction of pathogenic microorganisms.

The data in Table 8 and Table 9 are derived from the experimental data. The following formula was used:

$$\%=\frac{N_{0}-N}{N_{0}}\cdot 100$$

N_0_ = the initial number of microorganisms in the aqueous solution (colony-forming unit, CFU);

N = the final number of microorganisms after a contact time t (CFU).

In the case of the garlic–methanol biocide, it was found to be much more effective compared to the lavender–water biocide, even when tested at a bacterial load of coliform bacteria approximately 5 times higher than that used with the lavender–water biocide. With a volume of 5 mL of garlic–methanol biocide, total elimination of coliform bacteria was achieved after 60 min. When the volume of biocide was increased to 10 mL, total removal was achieved in just 5 min (Table 9).

It was found that the microbial activity and efficiency of the biocide depend on the initial sample’s microbial load, the volume of biocide used, and the contact time. The microbial load of the surface water was influenced by factors such as the water temperature, pH, dissolved oxygen, phytoplankton, etc.

Total inhibition of coliform bacteria in the water was achieved after adding 10 mL of garlic–methanol biocide for 5 min, with a 100% yield. In comparison, when using lavender–water biocide, the total inhibition of microorganisms was observed after adding 5 mL of biocide for 60 min.

## 4. Discussion

The most significant bioactive component in garlic is allicin, which is responsible for many of its medicinal properties. Allicin content in garlic has been found to be influenced by the geographical location of the harvesting site and genetic factors [34]. Although Burton et al. (2023) [35] stated that allicin is the only garlic component linked to its antimicrobial activity, a study by Balogun et al. (2023) [17] attributed the antimicrobial properties of garlic and onion extracts, used for preserving beef and chicken products, to their phenolic and flavonoid contents [17,36]. The extracts were found to have inhibitory effects against *Bacillus cereus*, *Staphylococcus aureus*, *Pseudomonas aeruginosa*, and *Salmonella typhimurium*, with lesser effects against *Escherichia coli*. El-Sayed et al. (2017) [37] found that the major components responsible for garlic’s antimicrobial properties were diallyl trisulfide and diallyl disulfide. In our study, the ^1^H NMR spectrum of the garlic–methanol extract indicates the presence of sulfur-containing organic compounds with antimicrobial activity against water pathogens. This indicates a general consensus regarding the antimicrobial properties of garlic extracts, though the active components may vary in composition and content.

At the same time, *Lavandula latifolia* is another medicinal plant known for its fungicidal, antibacterial, and antioxidant properties. These biological activities are attributed to its chemical composition, which includes classes of bioactive compounds such as flavonoids and terpenoids [38].

The biocides are inexpensive and easy to prepare. Preparation costs are low for both raw materials and labor. For example, 1 kg of garlic costs between 1 and 6 euros, while methanol costs around 4 euros per liter. With a methanol-to-garlic ratio of 5:1, the cost of one liter of extract would range from 21 to 26 euros. To this, energy costs for 24 h mixing using a mechanical stirrer (24 h), mechanical filtration on quantitative filter paper, and centrifugation, must be added. In total, the final price of one liter of biocide would be around 30 euros. For use, the biocide needs to be diluted 100-fold.

The obtained biocide can be effectively used in a wastewater treatment plant [39] before the biological treatment step. After this process, methanol can serve as an additional carbon source for bacteria in the activated sludge.

The very small amount of methanol in the biocide is not harmful. In a water treatment plant [40], disinfection with this type of biocide can be applied before the tertiary treatment stage with activated carbon.

The biocides do not require expensive raw materials or special synthesis methods. Given the ongoing global drought, every water resource is critical. These biocides can be applied in poor or remote areas, offering a practical solution for various water sources.

In this study, kinetic analysis plays a critical role for several key reasons. It enables a detailed evaluation of biocide efficacy by assessing how effectively biocides eliminate pathogenic microorganisms. This allows us to analyze the degradation rate and understand how quickly and efficiently each biocide acts under specific conditions. By applying a pseudo-first-order kinetic model, we can determine the degradation rates of these microorganisms, providing essential information about the time required to reduce their population to zero, if feasible. Moreover, kinetic analysis helps optimize both the biocide dose and contact time necessary to achieve maximum antimicrobial activity, which is crucial for practical applications in surface water treatments. This ensures that the minimal amount of biocide is used to achieve the desired outcomes. The kinetic model also facilitates the comparison of different biocides, such as lavender–water and garlic–methanol, under identical conditions, helping to identify the most effective biocide for surface water treatment. Additionally, kinetic analysis accounts for environmental factors like the temperature, pH, and the presence of other organic or inorganic substances, providing a deeper understanding of how antimicrobial activity varies across different environmental settings. It also plays a role in validating the mechanism of action of biocides, ensuring they behave as expected according to theoretical models.

The pseudo-first-order model, for instance, assumes that the reaction rate is proportional to the microorganism concentration, and validating this model enhances confidence in the predictability and effectiveness of the treatments. Additionally, kinetic analysis contributes to the development of new biocides by offering valuable insights into the mechanisms and efficiency of existing products, paving the way for modifications to chemical structures and the creation of biocides with enhanced properties.

Therefore, kinetic calculations and studies are fundamental to understanding and optimizing the use of garlic- and lavender-based biocides in surface water treatment, ensuring maximum efficiency in eliminating pathogenic microorganisms.

## 5. Conclusions

Biomimetic studies on garlic- and lavender-based biocides for surface water treatment leverage the natural antimicrobial properties of these plants to develop sustainable and effective water treatment solutions. These studies range from the extraction and characterization of active compounds to laboratory testing, field studies, and consideration of environmental impacts. The goal is to create natural biocides that are not only effective in reducing microbial contamination but also safe for the environment.

The two tested biocides have proven to be efficient in removing pathogens from surface waters. The synthesis method is simple, and the materials used are inexpensive and safe, both in terms of human health in case of ingestion and in terms of handling and storage.

After adding 1 mL of lavender–water biocide for 60 min, the inhibition yield of pathogenic microorganisms present in the water was 59.09%, while the garlic–methanol biocide yielded 40.86% under the same conditions.

After adding 5 mL of lavender–water biocide for 30 min, the inhibition yield of coliform bacteria present in the water was 51.85%, compared to the garlic–methanol biocide, which had a yield of 94.79% under the same conditions.

GC-MS analysis of the garlic–methanol biocide did not highlight the presence of compounds recognized as having antimicrobial activity. In the case of the FTIR analysis of garlic–methanol extract, the weak signal at wavelength of 1110.74 cm^−1^ was attributed to the extraction solvent. In the ^1^H NMR spectrum of the garlic–methanol extract, the group of signals recorded in the range of 4.33–4.29 ppm indicates the presence of organic compounds with sulfur. The FTIR spectrum of the lavender–water extract indicated the presence of linalool and linalyl acetate, which are bioactive in removing microorganisms from water.

## Figures and Tables

**Table 1:** Components of the surface waters.

Parameter	Value	Method
${NO}_{3}^{-}$ N	35 mg/L	SR ISO 7890/1-98
${SO}_{4}^{2-}$	15 mg/L	STAS 8061-70
Cl^−^	40 mg/L	STAS 6364-78
Na^+^	65.2 mg/L	STAS 3223/2-80
Ca^2+^	83.9 mg/L	SR EN ISO 7980:2002
K^+^	18.2 mg/L	STAS 3223/1-92
pH	7.2	SR EN ISO 10523:2012
Coliform bacteria	124 CFU/100 mL	SR EN ISO 9308-1: 02.2015

**Table 2:** Kinetic parameters of degradation with lavender–water biocide.

Kinetic Parameter	N_0_ = 124 CFU	N_0_ = 124 CFU	N_0_ = 124 CFU	N_0_ = 124 CFU
	V_biocide_ = 1 mL	V_biocide_ = 3 mL	V_biocide_ = 5 mL	V_biocide_ = 10 mL
k_obs_, min^−1^	0.0048	0.0142	0.0199	0.0486
R^2^	0.903	0.9841	0.9945	0.9427
r_0_, CFU/min	0.1584	0.3124	0.3781	1.3122
t_1/2_, min	144.41	48.81	34.83	14.26

**Table 3:** Kinetic parameters of degradation with garlic–methanol biocide.

Kinetic Parameter	N_0_ = 124 CFU	N_0_ = 124CFU	N_0_ = 124 CFU
	V_biocide_ = 1 mL	V_biocidE_ = 5 mL	V_biocidE_ = 10 mL
k_obs_, min^−1^	0.0114	0.1412	1.8316
R^2^	0.8294	0.976	0.9916
r_0_, CFU/min	1.0602	13.5552	227.12
t_1/2_, min	60.802	4.9089	0.3784

**Table 4:** One-way analysis of variance (ANOVA) test results for lavender–water biocide, V = 1 mL.

Source of Variation	Sum of Squares	Degrees of Freedom	Mean Squares	F Value	*p* Value
Between Groups	13,449.0	3	4483.0	192.82	8.47 × 10^−8^
Within Groups	186.0	8	23.25		
Total	13,635	11			

**Table 5:** One-way analysis of variance (ANOVA) test results for lavender–water biocide, V = 10 mL.

Source of Variation	Sum of Squares	Degrees of Freedom	Mean Squares	F Value	*p* Value
Between Groups	22,950.0	3	7650.0	244.8	3.3 × 10^−8^
Within Groups	250.0	8	31.25		
Total	23,200	11			

**Table 6:** One-way analysis of variance (ANOVA) test results for garlic–methanol biocide, V = 1 mL.

Source of Variation	Sum of Squares	Degrees of Freedom	Mean Squares	F Value	*p* Value
Between Groups	10,456.29	6	1742.71	70.11	1.22 × 10^−9^
Within Groups	348.0	14	24.86		
Total	10,804.29	20			

**Table 7:** One-way analysis of variance (ANOVA) test results for garlic–methanol biocide, V = 10 mL.

Source of Variation	Sum of Squares	Degrees of Freedom	Mean Squares	F Value	*p* Value
Between Groups	24,991.37	3	8330.46	9.29	1.87 × 10^−3^
Within Groups	10,756.75	12	896.40		
Total	35,748.12	15			

**Table 8:** Degradation yield of pathogenic microorganisms put in contact with lavender–water biocide, (a) V = 1 mL, (b) V = 3 mL, (c) V = 5 mL, (d) V = 10 mL.

Contact Time, min	η, % (a)	η, % (b)	η, % (c)	η, % (d)
0	0	0	0	0
15	9.09	22.72	31.57	48.14
30	21.21	27.27	47.36	51.85
60	32.05	59.09	68.42	96.29

**Table 9:** Degradation yield of fecal coliforms put in contact with garlic–methanol biocide, (a) V = 1 mL, (b) V = 5 mL, (c) V = 10 mL.

Contact Time, min	|, % (a)	|, % (b)	|, % (c)
0	0	0	0
1	4.30	39.58	91.93
5	12.90	52.08	99.19
10	21.50	65.62	99.99
15	34.40	87.5	99.99
30	37.63	94.79	99.99
60	40.86	99.98	99.99

## Data Availability

The data presented in this study are available on request from the corresponding author.
